# Ectopic pregnancy, its potential links to dementia risk and interactions with depression: insights from a nationwide cohort study

**DOI:** 10.3389/fpsyt.2024.1410685

**Published:** 2024-08-30

**Authors:** Chia-Yi Yao, Chi-Hsiang Chung, Wu-Chien Chien, Sung-Tao Li, Siou-Ting Lee, Chih-Chung Huang, Chuan-Chi Yang, Nian-Sheng Tzeng

**Affiliations:** ^1^ Department of Psychiatry, Tri-Service General Hospital, School of Medicine, National Defense Medical Center, Taipei, Taiwan; ^2^ Department of Medical Research, Tri-Service General Hospital, National Defense Medical Center, Taipei, Taiwan; ^3^ School of Public Health, National Defense Medical Center, Taipei, Taiwan; ^4^ Taiwanese Injury Prevention and Safety Promotion Association, Taipei, Taiwan; ^5^ Graduate Institute of Life Sciences, National Defense Medical Center, Taipei, Taiwan; ^6^ Department of Obstetrics and Gynecology, Tri-Service General Hospital, School of Medicine, National Defense Medical Center, Taipei, Taiwan; ^7^ Department of Obstetrics and Gynecology, Taoyuan Armed Forces General Hospital, Taoyuan, Taiwan; ^8^ Department of Psychiatry, Taoyuan Armed Forces General Hospital Hsinchu Branch, Hsinchu, Taiwan; ^9^ Department of Psychiatry, Taoyuan Armed Forces General Hospital, Taoyuan, Taiwan; ^10^ Counseling Center, National Defense Medical Center, Taipei, Taiwan

**Keywords:** ectopic pregnancy, dementia, vascular dementia, depression, antidepressant, national cohort study

## Abstract

**Background:**

Dementia poses a growing global mental health impact, with variations in prevalence by gender, possibly influenced by reproductive factors. Ectopic pregnancy (EP), known for its association with cardiovascular diseases and depression, which are also predictors of dementia, prompted an exploration of their interplay.

**Methods:**

Using Taiwan’s National Health Insurance Research Database, this nationwide cohort study examined 53,096 individuals to investigate the link between EP and dementia. Covariates included age, insured premiums, comorbidity by Charlson Comorbidity Index revised by excluding dementia, level of care, and residence. Surgical approaches, number of EP episodes, and dementia subtypes were considered in outcomes analysis using Cox regression.

**Results:**

Among 13,274 women diagnosed with EP, 791 developed dementia over a 15-year follow-up, particularly vascular dementia. Adjusting for the covariates, the adjusted sub-distribution Hazard Ratio (asHR) with competing risks was 1.644 (95% CI, 1.394–2.053; p < 0.001). For patients with more than one episode, it was even higher (asHR=1.670 [95% CI, 1.419–2.092; p < 0.001]). Post-ectopic depression, prevalent in 62.2% within four weeks, was associated with a greater dementia risk compared to those without (asHR=1.702 [95% CI, 1.444-2.125; p<0.001] vs. asHR=1.551 [95%CI, 1.310-1.937; p<0.001]). Antidepressant treatments showed a partial protective effect, reducing the increased risk by 14.7%.

**Conclusion:**

An EP history is linked to an earlier onset and a higher risk of overall dementia, VaD in particular, in a dose dependent manner, regardless of surgical intervention and stroke. Post-ectopic depression exacerbates dementia risk, while antidepressants offer partial protection. These findings underscore the potential benefit of screening and treating depression in women following EPs.

## Introduction

Dementia is a globally disabling disorder and should be acknowledged as a pandemic (1). In Taiwan, where the elderly demographic is burgeoning, dementia prevalence is estimated at 4.52% among individuals aged 65 and older. This number is expected to continue rising with the aging society (60). Gender disparities in dementia prevalence and outcomes have been noted, with women exhibiting a 1.17-fold higher age-standardized prevalence compared to men (2).

Women typically have a longer life expectancy than men, however, longevity itself may not explained the difference of prevalence of dementia between genders. Hormone factors, environmental factors, social roles and behaviors, and education opportunities might play a part in varying levels (3). Shorter cumulative endogenous estrogen exposure correlates with heightened all-types of dementia risk in women (4). Low levels of circulating and brain estrogen in women, and low testosterone in men, have been linked to an increased risk of AD. (5). ApoE4 alleles increase AD risk (5), and ApoE4 genotypes associated with higher fecundity (6). Past fertility is influenced by ApoE3/ApoE3 and ApoE3/ApoE2 genotypes, and having children had a significantly lower age of AD onset compared to those without children, but this phenomenon was not seen among ApoE4 carriers (61).

Loss of estrogen at menopause, appears to increase risk of AD, whereas estrogen treatments may be protective against AD (7), with cognitive benefits (8), and attenuating the heightened AD risk in women with ApoE4 genotype (9). Although there may not be a definite sex difference in the incidence of VaD (10), underlying sex differences in the pathophysiology are still possible: One study suggests that circulating estrogens might have a protective, anti-inflammatory effect, which preserve the vascular function in neuroinflammation, and might be relevant to VaD (11).

However, researches has shown that that both a high estrogenic or a high progestogenic environment can contribute to an increased risk of EP (12). Whether the hormone dysregulation and fluctuation during and after an ectopic pregnancy raise the risk of dementia or not, remained known. Pregnancy loss, including miscarriage, stillbirth, and induced abortion, has been associated with an increased risk of cardiovascular diseases, coronary heart disease (CHD), and stroke (13), all recognized risk factors for dementia (14–16). Ectopic pregnancy (EP), characterized by the implantation of an embryo outside the uterine endometrium, stands as the primary cause of maternal mortality during the first-trimester of pregnancy (17). Survivors often endure physical and psychological distress stemming from pain, bleeding, and the loss of an anticipated child. Moreover, a history of EP is linked to increased risks of premature cardiovascular events, cancer, and suicide-related mortality (18).

Following an EP, many women experience moderate to severe depression (19). An association between depressive symptoms and dementia has been observed, but the nature of their relationship is still debated. There are 5 possible hypotheses: 1) depression and dementia are commonly comorbid. 2) depression is a unadjusted reaction to dementia 3) depression impair the patient’s cognition and lead to a dementia-like clinical picture 4) depression is a risk factor of dementia 5) depression is a prodromal feature of dementia or the two disease shared the same mechanism (20). Given the potential for timely depression treatment to mitigate this progression (21), the long-term psychiatric implications for EP survivors warrant careful consideration.

Despite a prior nationwide cohort study links stillbirth, but not miscarriage, to dementia risk, EP has been notably absent from the investigation (22). To the best of our knowledge, there has been no nationwide research specifically focusing on the association between dementia risk and EP. In this study, we hypothesize that EP may be associated with an increased risk of dementia. Within a nationwide cohort of 53,096 women, we compare the risks of dementia, both overall and by subtype, between those with and without a history of EP. Additionally, we explore the potential influence of post-EP depression and the use of antidepressant treatment in these associations.

## Materials and methods

### Data sources

Data for our nationwide cohort study were extracted from the Taiwan National Health Insurance Research Database (NHIRD), which archives comprehensive data on ambulatory care, inpatient care, dental care, and prescription drugs availed by the insured and registration files. Enacted in 1995, the National Health Insurance (NHI) program ensures healthcare coverage for over 23 million individuals, representing 99% of the population. The diagnosis of one’s diseases was based on International Classification of Disease, Ninth Revision, and Clinical Modification (ICD-9-CM). The NHI Administration periodically conducts random reviews of ambulatory care visits and inpatient claims to validate diagnoses. Previous studies comprehensively document the program’s details (23, 24).

### Study population

This retrospective cohort study examined records from the NHIRD spanning 2000 to 2015, comprising 53,096 individuals in Taiwan. (refer to **Supplementary Figure S1**). The cohort comprised 13,274 female patients aged over 18 years diagnosed with ectopic pregnancy (EP). Exclusion criteria included pre-existing diagnoses of dementia or EP before 2000 and incomplete tracking information.

For each EP patient, three female controls with history of livebirth but without a history of EP, natural fetal loss, indeterminate loss, unspecified abortion, illegally induced abortion, or failed attempted abortion, were selected, matched using a 3-fold propensity score considering age and inclusion date. The ICD-9-CM codes are listed in **Supplementary Table S1**. Exclusion criteria were uniformly applied to both EP and control groups.

### Covariates

The covariates encompassed in the analysis consisted of age groups, geographic residence area, urbanization levels of residence, hospital classifications, and monthly income. We treat the age as a categorical variable in order to know at what age will dementia develop.

Urbanization levels were defined based on population size and various indicators related to political, economic, cultural, and metropolitan development. Level 1 was characterized by a population exceeding 1,250,000 with specific designations in political, economic, cultural, and metropolitan development. Level 2 included a population ranging from 500,000 to 1,249,999, playing a significant role in the political system, economy, and culture. Urbanization levels 3 and 4 were defined by populations between 149,999 and 499,999, and less than 149,999, respectively.

The collected data encompassed information on the number of medical visits due to EP, the utilization of surgery for treating EP, the diagnosis of depression within 4 weeks after EP, and the use of antidepressant treatment if post-ectopic depression was diagnosed.

### Comorbidity

The Charlson Comorbidity Index (CCI) is originally designed to categorize comorbid conditions for predicting long-term mortality. Extensive studies have demonstrated excellent inter-rater reliability and clinometric sensitivity across various medical conditions, with an observed correlation between higher CCI scores and increased mortality, making it a widely adopted tool for comorbidity risk adjustment in database studies.

This index classifies prior myocardial infarction and 19 other comorbidities into four groups based on their relative risk, with the weighted score indicating the patient’s risk of death (25). For our study, dementia has been excluded from the CCI, resulting in the creation of the Charlson Comorbidity Index revised (CCI_R).

### Study outcomes

Among the patients diagnosed with EP, we further document if they accepted surgical approaches or not. Basing on any medical visits beyond 180 days after the initial diagnosis, patients were grouped into having only one episode of EP and having more than one. Post-ectopic depression was defined as a diagnosis of depressive disorders within 4 weeks after the event, and at least three visits in a consecutive year.

Among those who presented with post-ectopic depression, we further document if they have antidepressant treatments or not. Strokes before and during the study are also recorded among the EP survivors, to exclude the possible causal effect on VaDs.

The list of ICD-9-CM codes for surgical interventions and depressive disorders, and Anatomical Therapeutic Chemical Classification codes of antidepressants, is in **Supplementary Table S1**.

All participants were observed until dementia diagnosis, NHIRD withdrawal, or the study’s end in 2015. A dementia diagnoses, classified into AD, VaD, and other forms of dementia, requires at least three visits in a consecutive year. The age at which dementia was first diagnosed was also recorded.

### Statistical analysis

SPSS software version 22 (SPSS Inc., Chicago, USA) facilitated the analysis. Categorical and continuous variables were assessed with χ2 tests and t-tests, respectively. The Fisher exact test compared categorical variables between cohorts. Multivariate Cox proportional hazards regression, with and without Fine & Gray’s competing risk model and Bonferroni correction, determined dementia risk (expressed as hazard ratio [HR] with 95% confidence interval [CI]). A sensitivity analysis excluding the diagnosis of dementia both within the first year and the first 5 years was conducted to avoid protopathic bias. Kaplan-Meier method and log-rank test assessed dementia risk disparity between EP subjects and controls.

While Kaplan-Meier and Cox models indicate the probability of surviving without dementia over time, they do not account for competing risks, such as death from ectopic pregnancy before developing dementia. The Fine-Gray competing risk model was developed to consider these competing risks, providing a better estimation of the actual risk.

Ectopic pregnancy, which has a mortality rate of up to 0.5 deaths per 1000 pregnancies (26), means that patients who die from it and its complications will not develop dementia. Therefore, we used Fine & Gray’s competing risk model to correct for this potential bias.

Significance was set at P value< 0.05 (2-tailed), with data analysis overseen by a statistician. When performing multiple statistical tests, we used the Bonferroni correction to adjust p-values due to the increased risk of a type I error.

## Results

### Study cohort characteristics

The enrollment process for patients is delineated in **Supplementary Figure S1**, with 13,274 women diagnosed with EP and 39,822 women comprising the control group. Over the follow-up period, 791 cases of dementia were identified among EP patients. **Table 1** presents the baseline characteristics of the study population.

**Table 1:** Characteristics of study in the baseline.

Ectopic pregnancy	With		Without		*P*
Variables	n	%	n	%	
**Total**	13,274		39,822		
**Age (years)**	41.12 ± 15.26		41.36 ± 15.78		0.126
**Age groups (yrs)**					0.999
18 - 19	290	2.18	870	2.18	
20 - 24	131	0.99	393	0.99	
25 - 29	424	3.19	1,272	3.19	
30 - 34	1,523	11.47	4,569	11.47	
35 - 39	3,956	29.80	11,868	29.80	
40 - 44	4,832	36.40	14,496	36.40	
≧ 45	2,118	15.96	6,354	15.96	
**Insured premium (NT$)**					< 0.001
< 18,000	7,012	52.83	20,149	50.60	
18,000 - 34,999	4,569	34.42	14,278	35.85	
≧ 35,000	1,693	12.75	5,395	13.55	
**CCI_R**	1.01 ± 1.03		0.95 ± 0.97		< 0.001
**Location**					< 0.001
Northern Taiwan	3,870	29.15	12,065	30.30	
Middle Taiwan	3,691	27.81	11,143	27.98	
Southern Taiwan	3,785	28.51	10,427	26.18	
Eastern Taiwan	1,542	11.62	3,875	9.73	
Outlets islands	386	2.91	2,312	5.81	
**Urbanization level**					< 0.001
1 (The highest)	3,813	28.73	12,031	30.21	
2	3,980	29.98	13,798	34.65	
3	2,516	18.95	5,979	15.01	
4 (The lowest)	2,965	22.34	8,014	20.12	
**Level of care**					< 0.001
Hospital center	4,877	36.74	13,392	33.63	
Regional hospital	5,738	43.23	14,272	35.84	
Local hospital	2,659	20.03	12,158	30.53	

P: Chi-square/Fisher exact test on category variables and t-test on continue variables.

The distribution of insured premiums reveals a significant difference (p < 0.001), with a higher proportion of individuals with EP falling into the <18,000 NT$ premium category (52.83%) compared to those without (50.60%).

The Charlson Comorbidity Index revised (CCI_R) differs significantly between the two groups (p < 0.001), with those experiencing EP having a slightly higher mean value (1.01 ± 1.03) compared to those without (0.95 ± 0.97).

In the EP group, significant differences in distribution across regions are observed (p < 0.001), with more women living in Southern and Eastern Taiwan. Urbanization level is also significantly lower in the disease group (p < 0.001). Women with EP are more likely to seek treatment at regional hospitals and hospital centers (p < 0.001).

### Kaplan–Meier curves for the cumulative incidence of dementia in patients with EP

**Figure 1:**
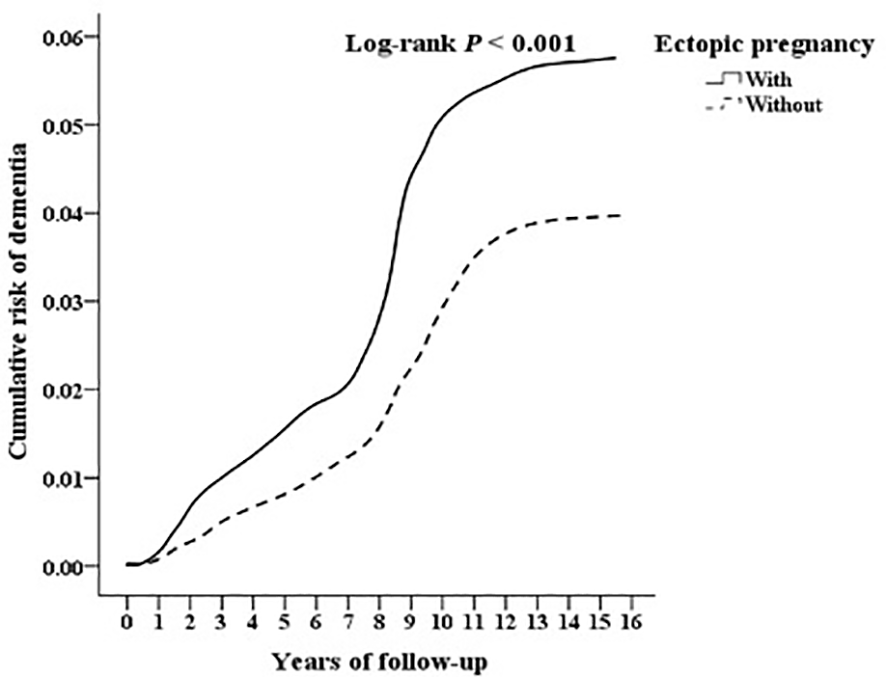
Kaplan-Meier for cumulative risk of dementia aged 18 and over in women stratified by ectopic pregnancy with log-rank test.

The cumulative incidence of dementia was notably higher in the EP group compared to controls (555.88 versus 360.84 per 10^5^ person-years, respectively; **Supplementary Table S1**), as evidenced by the log-rank test (p < 0.001; **Figure 1**, **Table 2**).

**Table 2:** The tracking years of dementia after ectopic pregnancy.

Ectopic pregnancy	With (n = 13.274)	Without (n = 39,822)	Log-rank *P*
In the tracking of x year(s)	Numbers of dementia		
**1**	49	108	0.986
**2**	101	219	0.012
**3**	148	327	< 0.001
**4**	199	435	< 0.001
**5**	247	546	< 0.001
**6**	301	651	< 0.001
**7**	345	762	< 0.001
**8**	391	866	< 0.001
**9**	446	985	< 0.001
**10**	498	1,094	< 0.001
**11**	543	1,203	< 0.001
**12**	592	1,310	< 0.001
**13**	640	1,425	< 0.001
**14**	697	1,539	< 0.001
**15**	743	1,644	< 0.001
**16**	791	1,752	< 0.001

### Hazard ratio analysis of dementia in patients with EP

Cox regression analysis, as detailed in **Table 3**, revealed a significantly elevated risk of dementia among EP patients. The crude HR was 2.186 (95% CI, 1.798–2.701; p < 0.001), while the adjusted HR (aHR) without competing risks was 1.604 (95% CI, 1.375–1.982; p < 0.001). Considering competing risks, the adjusted sub-distribution HR (asHR) was 1.644 (95% CI, 1.394–2.053; p < 0.001), indicating a persistent association between EP and dementia risk after adjusting for confounding variables.

**Table 3:** Factors of dementia by using Cox regression with/without Fine & Gray’s competing risk model.

	*No competing risk*								*Competing risk*			
Variables	Crude HR	95%CI	95% CI	*P*	aHR	95% CI	95% CI	*P*	asHR	95% CI	95% CI	*P*
Ectopic pregnancy												
Without	Reference				Reference				Reference			
With	2.186	1.798	2.701	< 0.001	1.604	1.375	1.982	< 0.001	1.644	1.394	2.053	< 0.001
Age group (yrs)												
18 - 19	–	–	–	–	–	–	–	–	–	–	–	–
20 - 24	0.000	–	–	0.999	0.000	–	–	0.999	0.000	–	–	0.999
25 - 29	0.000	–	–	0.999	0.000	–	–	0.999	0.000	–	–	0.999
30 - 34	0.000	–	–	0.999	0.000	–	–	0.999	0.000	–	–	0.999
35 - 39	0.000	–	–	0.999	0.000	–	–	0.999	0.000	–	–	0.999
40 - 44	Reference				Reference				Reference			
≧ 45	8.031	3.999	15.975	< 0.001	5.648	3.183	13.982	< 0.001	5.789	3.271	14.521	< 0.001
Insured premium (NT$)												
< 18,000	Reference				Reference				Reference			
18,000 - 34,999	0.875	0.509	1.089	0.427	0.915	0.628	1.134	0.372	0.937	0.637	1.178	0.367
≧ 35,000	0.672	0.379	0.911	0.006	0.742	0.482	1.098	0.511	0.760	0.489	1.139	0.509
**CCI_R**	1.292	1.198	1.378	< 0.001	1.241	1.172	1.325	< 0.001	1.272	1.189	1.374	< 0.001
Location												
Northern Taiwan	Reference				**Multicollinearity with urbanization level**				**Multicollinearity with urbanization level**			
Middle Taiwan	0.897	0.311	1.824	0.672	**Multicollinearity with urbanization level**				**Multicollinearity with urbanization level**			
Southern Taiwan	0.975	0.480	2.097	0.583	**Multicollinearity with urbanization level**				**Multicollinearity with urbanization level**			
Eastern Taiwan	0.683	0.279	1.276	0.813	**Multicollinearity with urbanization level**				**Multicollinearity with urbanization level**			
Outlets islands	0.370	0.022	10.704	0.974	**Multicollinearity with urbanization level**				**Multicollinearity with urbanization level**			
Urbanization level												
1 (The highest)	2.046	1.506	2.586	< 0.001	1.672	1.214	2.090	< 0.001	1.717	1.234	2.169	< 0.001
2	1.782	1.311	2.178	< 0.001	1.597	1.103	1.996	< 0.001	1.637	1.123	2.072	< 0.001
3	1.332	1.082	1.672	0.009	1.045	0.783	1.488	0.218	1.074	0.791	1.548	0.206
4 (The lowest)	Reference				Reference				Reference			
Level of care												
Hospital center	2.465	1.808	3.012	< 0.001	1.986	1.240	2.786	< 0.001	2.039	1.258	2.899	< 0.001
Regional hospital	1.560	1.124	1.986	< 0.001	1.303	1.050	1.672	0.025	1.334	1.065	1.732	0.018
Local hospital	Reference				Reference				Reference			

HR, hazard ratio; CI, confidence interval; aHR, Adjusted HR: Adjusted variables listed in the **Table 3**.

asHR, Adjusted Subdistrubtion Hazard ratio: Adjusted for the variables listed in the **Table 3**; Competing variable: all-caused mortality.

Despite significant variations in insured premiums, CCI_R, urbanization level, and level of care among individuals with EP at baseline (as indicated in **Table 1**), the multivariate Cox proportional hazards regression revealed that the EP cohort bore a heightened risk of all forms of dementia compared to the control group. This elevated risk persisted even after accounting for the aforementioned variables.

In both study and comparison cohort, the targeted event of dementia happened after the age 40. In the **Supplementary Table S2**, we found there is a significant difference in the average age at which dementia was first diagnosed among women who had EP and women who did not, indicating that EP was not only associated with an increased risk, but also an earlier onset of dementia.

**Figure 2:**
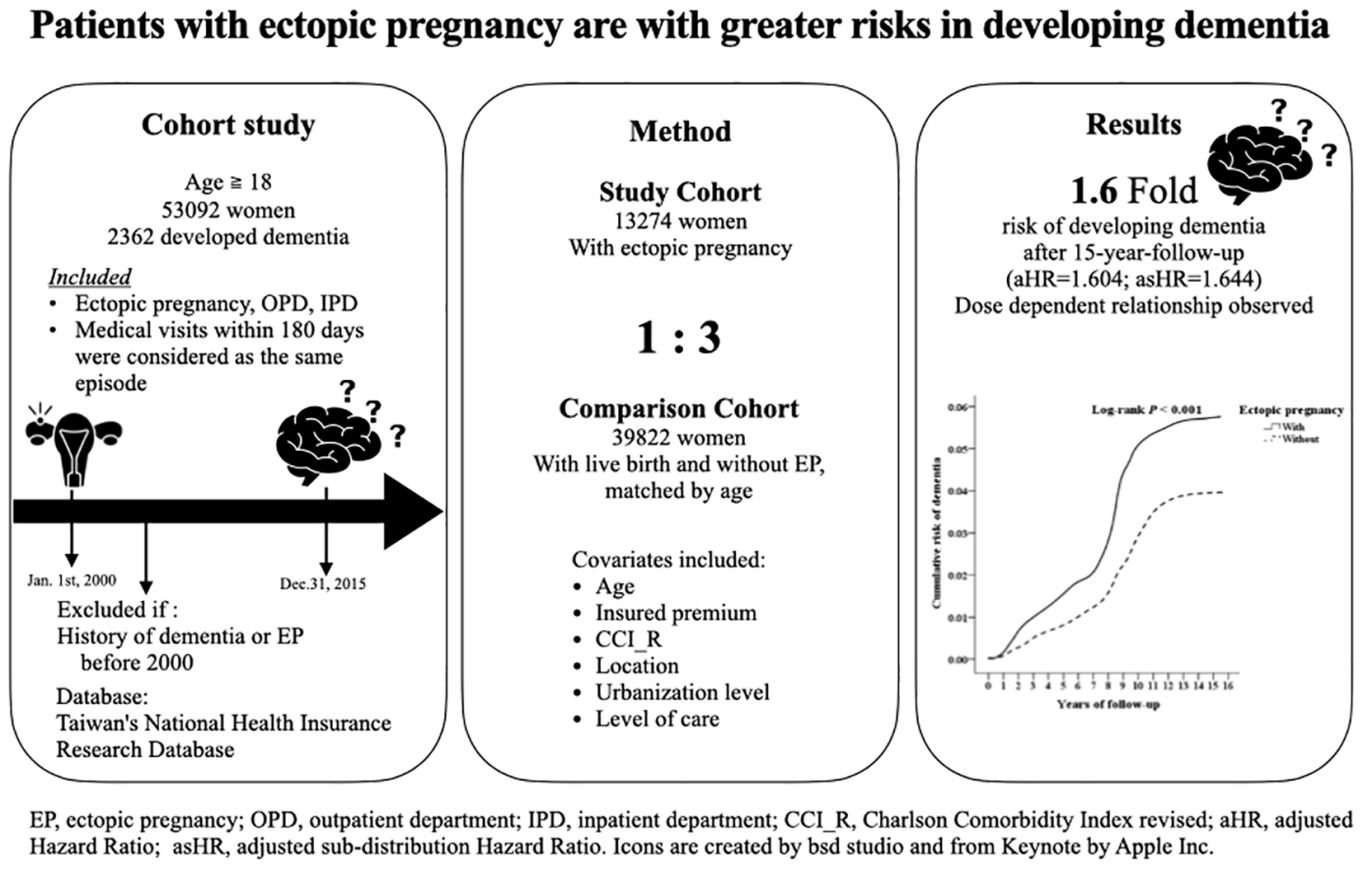
A graphic summary of our study.

A graphic abstract of the results is as shown in **Figure 2**.

### Subgroup analysis of dementia in EP cohort and controls

In **Table 4**, we additionally investigate the impact of variables such as the number of EP episodes, treatment modalities, the occurrence of post-ectopic depression, and the prescription of antidepressants for individuals experiencing post-ectopic depression on the subsequent risk of dementia in the EP cohort.

**Table 4:** Factors of dementia among different EP subgroups by using Cox regression with/without Fine & Gray’s competing risk model and Bonferroni correction for multiple comparisons.

					*No competing risk*				*Competing risk*			
Ectopic pregnancy subgroups	Populations	Events	PYs	Rate	aHR	95% CI	95% CI	*P*	asHR	95% CI	95% CI	*P*
Without ectopic pregnancy	39,822	1,572	435,652.39	360.84	Reference				Reference			
With ectopic pregnancy	13,274	791	142,297.78	555.88	1.604	1.375	1.982	< 0.001	1.644	1.394	2.053	< 0.001
Episode = 1	10,616	630	113,802.93	553.59	1.597	1.368	1.977	< 0.001	1.638	1.390	2.048	< 0.001
Episodes ≧ 2	2,658	161	28,494.85	565.01	1.631	1.399	2.018	< 0.001	1.670	1.419	2.092	< 0.001
Without surgery	11,601	690	124,355.57	554.86	1.602	1.374	1.976	< 0.001	1.642	1.392	2.048	< 0.001
With surgery	1,673	101	17,942.21	562.92	1.611	1.380	1.997	< 0.001	1.650	1.398	2.071	< 0.001
Without post-ectopic depression	5,013	280	53,431.97	524.03	1.512	1.296	1.867	< 0.001	1.551	1.310	1.937	< 0.001
With post-ectopic depression	8,261	511	88,865.81	575.02	1.660	1.421	2.051	< 0.001	1.702	1.444	2.125	< 0.001
Without antidepressants	1,035	76	11,513.72	660.08	1.905	1.634	2.355	< 0.001	1.953	1.657	2.441	< 0.001
With antidepressants	7,226	435	77,352.09	562.36	1.623	1.390	2.004	< 0.001	1.666	1.408	2.079	< 0.001
Without stroke	9,158	480	98,174.25	488.93	1.411	1.209	1.743	< 0.001	1.443	1.226	1.806	< 0.001
With stroke	4,116	311	44,123.53	704.84	2.034	1.742	2.513	< 0.001	2.085	1.769	2.603	< 0.001

PYs, Person-years; Rate: per 100,000 PYs, aHR, Adjusted Hazard ratio; Adjusted for the variables listed in **Table 3**, CI, confidence interval.

asHR, Adjusted Subdistrubtion Hazard ratio; Adjusted for the variables listed in **Table 3**; Competing variable: all-cause mortality.

A dose-dependent relationship was observed between the number of EP episodes and dementia risk, with higher risks associated with recurrent episodes. (asHR=1.638, 95% CI, 1.390-2.048; P<0.001 versus asHR=1.670, 95% CI, 1.419-2.092; P<0.001). The increased risks are similar between patients who did or did not undergo surgical interventions.

Notably, depression can be found in 62.2% women diagnosed with EP within 4 weeks. Those who suffered from post-ectopic depression have greater increased risk of developing dementia, compared with those who did not. (asHR=1.551, CI, 1.310-1.937; P<0.001 versus asHR=1.702, CI, 1.444-2.125; P<0.001) Thankfully, antidepressant treatment to such mental crisis showed partial protective effect by lowering 14.7% of increased risk. (asHR=1.953, CI, 1.657-2.441; P<0.001 versus asHR=1.666, CI, 1.408-2.079; P<0.001).

People who were ever exposed to strokes, including before and during the study, are seen as high as 31% among EP survivors in our study. Although those who ever suffered from stroke have greater increased risk of developing dementia, compared with those who did not (asHR=2.085, CI, 1.769-2.603; P<0.001 versus asHR=1.433, CI, 1.226-1.806; P<0.001), EP survivors without strokes remained elevated risks of dementia.

### Sensitivity analysis of the risk of dementia in patients with ectopic pregnancy

Patients with EP were linked to an elevated risk in overall dementia, in comparison to the control group. Even after excluding individuals diagnosed with dementia within the first year and the initial 5 years, the subjects with EP remained associated with an overall increased risk of dementia (**Table 5**).

**Table 5:** Sensitivity analysis for factors of dementia subgroups by using Cox regression with/without Fine & Gray’s competing risk model and Bonferroni correction for multiple comparisons.

Ectopic pregnancy	With			Without *(Reference)*			*No competing risk*				*Competing risk*			
Sensitivity analysis	Events	PYs	Rate	Events	PYs	Rate	aHR	95% CI	95% CI	*P*	asHR	95% CI	95% CI	*P*
**Overall**	791	142,297.78	555.88	1,572	435,652.39	360.84	1.604	1.375	1.982	< 0.001	1.644	1.394	2.053	< 0.001
In the first year excluded	742	134,430.21	551.96	1,464	404,413.25	362.01	1.532	1.310	1.924	< 0.001	1.578	1.325	1.988	< 0.001
In the first 5 years excluded	544	108,950.04	499.31	1,026	297,032.14	345.42	1.450	1.238	1.816	< 0.001	1.493	1.257	1.870	< 0.001

PYs, Person-years; Rate: per 100,000 PYs, aHR, Adjusted Hazard ratio; Adjusted for the variables listed in **Table 3**, CI, confidence interval.

asHR, Adjusted Subdistrubtion Hazard ratio; Adjusted for the variables listed in **Table 3**; Competing variable: all-caused mortality.

### The types of dementia developed in EP cohort and controls

**Supplementary Table S3** delineates dementia subgroup analysis within the EP cohort, demonstrating a significantly higher risk of overall dementia compared to controls. Vascular dementia (VaD) exhibited the most pronounced elevation in risk among AD, VaD, and other dementia subtypes within the EP cohort (asHR=1.848, 95% CI, 1.567-2.309; p < 0.001).

## Discussion

To our knowledge, this is the first study to examine national data on cognitive outcomes in women with EP history. Dementia incidence, VaD in particular, was significantly higher in EP cases than controls. The results are remained significant after sensitivity tests excluding the dementia diagnosis in the first year and first 5 years after EP. Although patients who have had expose to EP and strokes had higher risk of dementia than patients expose to only EP, the risk of dementia is higher in the patients without strokes. Patients suffered from EPs are also significantly younger than controls when they get the dementia diagnosis. Post-ectopic depression, prevalent in 62.2%, heightened dementia risk, with partial mitigation through antidepressant treatment.

### Comparison with previous studies

Reproductive history and the risk of mental disorders in later life had been studied in many aspects. In a multicenter prospective cohort study, (19) 908 women with and without miscarriages and EP within 1 month was enrolled and follow their anxiety, depression and post-traumatic stress symptoms. Higher portions (7%) of women with EP was found to present with moderate to severe depression, defined by a score ≧ 11 in the Hospital Anxiety and Depression Scale, in comparison with viable pregnant group (2%) after 1 month and remains at clinically significant level at 9 months (11%). Our findings are consistent with this trend but in a much larger scale, with 62.2% women developing major depression disorder, depressive episode of bipolar disorder, neurotic depression, adjustment disorder with depressed mood, prolonged depressive reaction, adjustment disorder with mixed anxiety and depressed mood, or depressive disorder, not elsewhere classified, and 54.3% being prescribed antidepressants. The differences may be partially explained by the potentially under-report of severity in the self-reported nature in the previous study. Cultural differences, particularly the societal emphasis on family lineage and support provision in Asia, may also contribute to these disparities (27). There haven’t been studies focusing on EP and the associations with dementia.

In a Danish nationwide cohort study enrolling 1,243,957 women, Basit et al. (22) observed that a previous occurrence of stillbirth correlated with nearly a twofold rise in the overall likelihood of developing dementia. This association’s potency seemed consistent across various ages of dementia onset, and the degrees of connection between stillbirth and distinct dementia subtypes did not exhibit statistically significant differences.

The connection may be attributed to shared mechanisms involving vascular pathology and endothelial dysfunction. These mechanisms could contribute to inadequate placental implantation during pregnancy, leading to pregnancy losses, and create a conducive environment for dementia later in life. Existing research associating pregnancy loss with vascular diseases supports the plausibility of links between complications related to vascular pathology during pregnancy and dementia, especially VaD, possibly via increased risk of cerebral infarction (28). Notably, in our study, an increased risk of dementia is observed even in EP survivors who have never developed strokes.

### Vascular pathology and endothelial dysfunction

Vascular pathology and endothelial dysfunction are also found to be contributory to the pathogenesis of EP. It is estimated that 95% of all EP occurs in the fallopian tubes (17). Impaired embryo-tubal transport leading the retention of the embryo within the fallopian tube may work in combination with the changes in the tubal environment that facilitate early implantation to cause such condition (29). Successful implantation relies on a supportive vascular network. Ectopic pregnancies necessitate a favorable blood supply and exhibit angiogenesis at implantation sites. Vascular endothelial growth factor (VEGF) may be the pro-angiogenic factor responsible for the implantation and placentation of a tubal EP, supporting by the evidence that upregulated mRNA expression of VEGF and its receptors, including KDR and flt-1, at the implantation site with ectopic gestation compared to the non-implantation site (30). In addition, serum VEGF level is positively correlated to the depth of trophoblastic invasion into the tube (31). Aberrant VEGF signaling is also believed to be the underlying the angiogenic dysfunction in AD (32).

### Dysregulation of miRNAs

MicroRNAs (miRNAs) are single-stranded RNA molecules, typically 19-24 nucleotides in length, that play a role in regulating post-transcriptional gene expression. An increased serum level of miR-323-3p has been identified as a stable biomarker capable of differentiating EP from spontaneous abortion and viable intrauterine pregnancy (33). Furthermore, miR-323-3p has demonstrated the ability to regulate amyloid precursor protein *in vitro* and under physiological conditions in cells (34), suggesting potential involvement in the pathogenesis of AD.

The miR-323-3p, miR-149 and miR-424 were found to be dysregulated in the cortex of patients with AD (35). Among them, dysregulation of miRNA-149 and miR-424 are also found to be linked to the estrogen receptor and progesterone receptor signaling pathways in women with ongoing tubal EP and are differentially expressed in the implantation site compared to the non-implantation site. The change may be count for the altered tubal environment that traps embryo to initiate abnormal implantation (36).

### Systemic inflammatory reaction

EP triggers a systemic inflammatory response, marked by increased inflammatory cytokines in the implantation region and systemic circulation (29). The Systemic Immune-Inflammation Index (SII), a gauge of the body’s immune-inflammatory response (37), exhibits a linear correlation with trophoblastic infiltration depth and serum β-hCG in EP (38). Dysregulation of chemokine Bv8/prokineticin 2 (PROKR2) is implicated as a risk factor, playing a crucial role in tubal EP pathogenesis (39). PROKR2, involved in inflammatory and neuroinflammatory conditions, contributes to β-amyloid toxicity, with AD patients displaying approximately twofold increased serum PROK2 levels (40).

### Oxidative stress status

Ectopic pregnancies may be linked to high oxidative stress (41). Reactive oxygen species (ROS) play a role in various processes related to human reproduction, such as folliculogenesis, oocyte maturation, ovulation, and fetoplacental development (42). However, when the balance between prooxidants and antioxidants is disrupted, and ROS levels exceed the capacity of intracellular antioxidant systems, it can lead to oxidative stress.

High oxidative stress may affect the fallopian tubes, causing dysfunction in the tubal epithelium and disrupting embryo transport. This disruption can increase the risk of tubal ectopic pregnancy (42).

The brain is also sensitive to oxidative stress due to its high oxygen demand, the susceptibility of its fatty acids to peroxidation, and limited antioxidant defenses. Therefore, it is not surprising that oxidative stress is involved in the development of both AD and VaD (43).

### The role of Apolipoprotein E polymorphisms

Astrocytes primarily express ApoE variants in the brain. These cells play critical roles in amyloid deposition, Tau phosphorylation, lipid distribution, cerebrovascular function, and mitochondrial dysfunction. Possession of the ApoE4 allele increases the risk of both AD and VaD (11).

In the ovary, the thecal cells and granulosa cells need ApoE for cholesterol transport (44). ApoE gene is 100-fold up-regulated during the implantation window and is highly expressed in the endometrium (45). There is also an association found between APOE gene alleles (ApoE2 and ApoE4) and altered lipid profiles that can negatively impact both the mother and fetus. This suggests that the ApoE genotype may influence a woman’s ability to carry a viable pregnancy (46).

A systematic review and meta-analysis showed women carrying the ApoE4 allele displaying a higher risk of recurrent pregnancy loss compared with those carrying the ApoE2 and ApoE3 alleles. These associations were restricted to the Asian population. (47). Further studies on ApoE genotypes and ectopic pregnancy are needed to know if there are shared mechanism via ApoE between dementia and ectopic pregnancy.

### Depression and dementia

Previous studies showed that depression was associated with increased risk of dementia. The risk factor hypothesis and the prodrome feature/shared mechanism hypothesis are supported by strongest evidence. Several studies show that depression before the age of 60 is significantly associated with an increased risk of developing dementia (20). One study (48) found that the risk of dementia, including all-cause dementia and Alzheimer’s disease, increases with the number of episodes of elevated depressive symptoms. All these above supports the hypothesis that depression may be a risk factor for dementia.

Meanwhile, a systematic review and meta-analysis found no link between depression or depressive symptoms and amyloid-beta levels detected by positron emission tomography (PET), cerebrospinal fluid, or plasma. However, a subgroup analysis in the same study found a connection between plasma amyloid-beta and depression or depressive symptoms in people with cognitive impairment. (49). Also, a 28-year follow-up study also revealed that only late-life, not midlife, depressive symptoms are linked to a higher risk of dementia. This suggests that depressive symptoms are a prodromal feature of dementia or that they share common causes with dementia (50).

It is hypothesis that there are mechanisms linking depression and dementia. These pathways include: 1) vascular pathology; 2) changes in glucocorticoid steroids and hippocampal atrophy; 3) increased β-amyloid plaque deposition; 4) inflammation; and 5) nerve growth factors or neurotrophins deficiency (20).

In our study, dementia risks were found to be higher in women with post-ectopic depression. This is not a diagnosis listed in the fifth edition of the Diagnostic and Statistical Manual of Mental Disorders (DSM-5). In consideration of the specifier “peripartum onset” of major depressive disorder was definite as an episode occurs during pregnancy or within 4 weeks of delivery, we identify a depressive episode happening within 4 weeks after the diagnosis of an EP to be “post-ectopic depression.” Fortunately, this risk can be partially lessened, although cannot reverse, by antidepressant treatment. Furthermore, antidepressant use is not associated with increased risk of EP (51).

Women with EP exhibited lower insured premiums. Previous research suggests that psychosocial factors like educational level, occupational attainment, income, and involvement in social and mental activities are associated with dementia risk. Therefore, potential confounding factors could contribute to this observed difference (52).

Our study supports post-ectopic depression to be a risk factor of dementia. However, we are not sure whether post-ectopic depression can be a prodromal feature of dementia, or whether a third factor causes them both. Future studies exploring the life course in such population will be valuable to a better understanding of the disease nature of both diseases.

### Ectopic pregnancy and the risk of stroke

In our study, 31% of EP survivors had strokes before or during the study, which is much higher than the prevalence of 5.26% found in a previous study targeting Taiwanese among the population aged 45-54 (53). An association of risk of stroke is therefore suspected among people with EP.

There is a case report on ischemic stroke coming along a ruptured ectopic pregnancy but the epidemiology data of such condition is lacking (54).

The reported risks of ischemic stroke, intracerebral hemorrhage, cerebral venous thrombosis, and subarachnoid hemorrhage during pregnancy vary across different studies. These variations may be due to differences in whether the studies divided pregnancy into trimesters, included only inpatients or outpatients as well, used different diagnostic systems, and the range of diseases they considered. Some studies report no increased risk during the 9 months of pregnancy except for a high risk in the 2 days prior and 1 day postpartum (55), while others report up to a threefold increase. However, there is a consensus that the risk peaks around delivery, from the third trimester to up to 12 weeks postpartum (56, 57). Notably, data suggest that rates of pregnancy-related stroke have been increasing in the US and Canada in recent years, may also be a reason why the data mixed. Reasons for increasing pregnancy-related stroke may include advancing maternal age at the time of birth and the increasing prevalence of traditional cardiovascular risk factors, and other risk factors, as well, such as hypertensive disorders of pregnancy, migraine, and infections (57).

Risk factors associated with pregnancy-related stroke can be roughly classified into cardiovascular, pregnancy-related and other factors. The first includes older age, obesity, smoking, hyperlipidemia, chronic hypertension, and heart disease. Pregnancy-related factors include hypertensive disorders of pregnancy, hypercoagulable states, postpartum cardiomyopathy, postpartum angiopathy and amniotic fluid embolus (56). Other risk factors include migraine and infections (57). However, we do not know if ectopic pregnancy is associated with a higher risk of stroke compared to a viable pregnancy.

A history of pregnancy-related stroke was not suggested to be a contraindication for subsequent pregnancy (57).

### Clinical implication

For survivors of ectopic pregnancy (EP), especially those with recurrent EPs, it is beneficial to screen for depressive symptoms, particularly during the first four weeks after the EP. Prescribing antidepressants may help protect against the risk of dementia. Additionally, evaluating cardiovascular risk and obtaining a history of strokes in women who have experienced EP can identify those who need secondary preventions from strokes, which are related to an acerbated elevation in risk of dementia. A comprehensive, multidisciplinary approach is recommended to optimize the management of women with EP.

### Limitations

In our study, all patients with dementia, both in EP and control group, get their first diagnosis of dementia before the age of 65, compatible with early-onset dementia. This type of dementia can have more severe psychosocial consequences, as it affects individuals during their peak career years and while they have significant caregiving duties. Considering the fact that dementia is more commonly seen in people aged 65 or older, selection bias may be present.

Also, although we have followed up the patients for as long as 15 years, it may not be sufficient for senile dementia, such as AD to fully developed. This may explain why AD counts only a lesser proportion in our study than in the general population. A longer follow-up study allowing more understanding for EP and the risk of senile dementia will be helpful in knowing more about the disease nature of both EP and dementia.

This study has some other limitations to acknowledge. Firstly, since dementia cases were identified through claimed data, information on severity, stage, or impact on caregivers were unavailable. Secondly, residual confounding factors like education, genetics, and psychosocial elements were not accounted for. Nonetheless, the study encompasses Taiwan’s hospitals and >99% of its population over 15 years, enhancing data validity. Additionally, AD is reported as the most common cause (40-60% of all dementias), followed by VaD (20-30%), and mixed or other dementias (7-15%) in Taiwan (58, 59). However, in our study, most dementias were vascular and other types. One possible explanation for this discrepancy is that some of the other dementias in our study might actually be ADs. Another possibility is that clinicians might categorize dementias with a progressive course and no evidence of previous cerebrovascular events into the “other” category instead of AD.

### Conclusion

The research underscores the importance of prioritizing the long-term cognitive and mental health support for women following EP. Sensitivity analysis showed that among Taiwanese women, EP correlates with an earlier onset and an elevated overall dementia risk in a dose-dependent manner, particularly if post-ectopic depression occurred. The increased risk remained among those who did not expose to strokes. However, the administration of antidepressants appears to mitigate this risk, although the specific classes, dosages, and treatment durations were not explored in this study. It’s worth noting that the study primarily focused on East Asian women, limiting the generalizability of findings to other ethnic groups. Further evaluations are needed using randomized clinical trials or observational studies in population with a border race diversity.

## Data Availability

The data on the study population that were obtained from the NHIRD (https://nhird.nhri.org.tw/en/index.html) are maintained in the NHIRD (https://nhird.nhri.org.tw/). The NHRI is a nonprofit foundation established by the government. Only citizens of Taiwan who fulfill the requirements of conducting research projects are eligible to apply for the NHIRD. The use of the NHIRD is limited to research purposes only. Applicants must follow the Computer-Processed Personal Data Protection Law (https://www.winklerpartners.com/?p=987) and the related regulations of the National Health Insurance Administration and NHRI, and an agreement must be signed by the applicant and their supervisor upon application submission. All applications are reviewed for approval of data release.
