# Supplementary material for: Ectopic pregnancy, its potential links to dementia risk and interactions with depression: insights from a nationwide cohort study
Source: Front Psychiatry. 2024 Aug 30;15:1410685. doi: 10.3389/fpsyt.2024.1410685 (PMC11392761; doi:10.3389/fpsyt.2024.1410685)
Supplement: Supplementary file 1 [file DataSheet1.docx]

**Supplementary Materials**

Figure S1. The flowchart of study


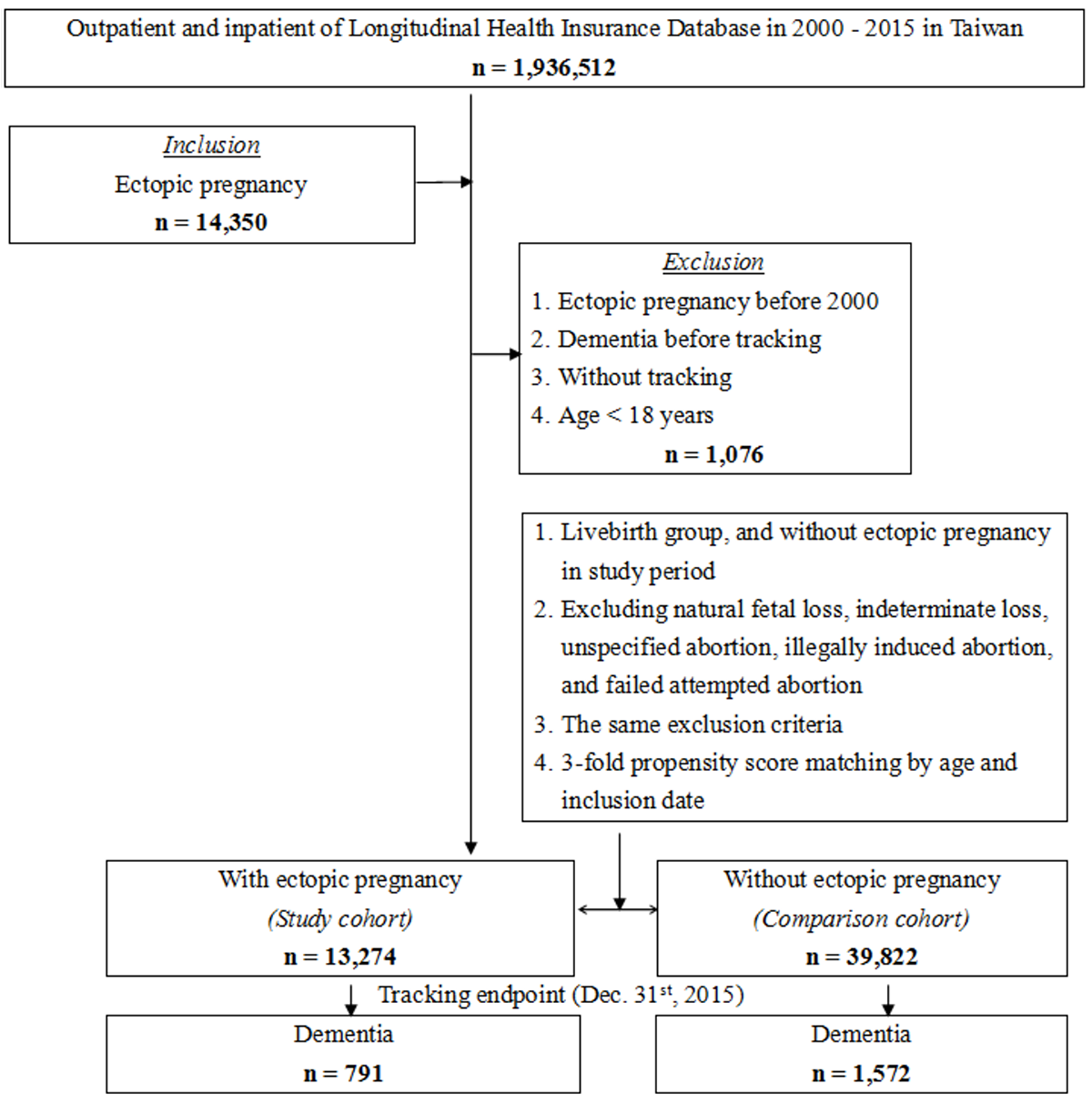


**Table S1 Abbreviation,** **The International Classification of Diseases, 9th Revision, Clinical Modification, Anatomical Therapeutic Chemical Codes, and Definitions**

|  | **Abbreviation** | **ICD-9-CM/ ATC-Code / Definitions** |
| --- | --- | --- |
| **Stduy population:** Ectopic pregnancy |  | 633; occurring within a 180-day period were considered to be part of the same episode |
| **Excluding:** |  |  |
| Natural fetal loss |  | 630, 631, 633, 634, 656.4 |
| Indeterminate loss |  | 632 |
| Unspecified abortion |  | 629.81, 635, 637, 639 |
| Illegally induced abortion |  | 636 |
| Failed attempted abortion |  | 638 |
| **Events:** Dementia |  | 290, 331.0; Medical visits ≧ 3; Diagnosed by neurologist or psychiatrist |
| Alzheimer's disease | AD | 331.0 |
| Vascular dementia | VaD | 290.4 |
| Other degenerative dementia | Others | 290.x except 290.4 |
| **Covariates:** |  |  |
| Surgical approaches to ectopic pregnancy | Surgery | OP66.01, OP66.02, OP66.62, OP74.3 |
| Salpingotomy |  | OP66.01 |
| Salpingostomy |  | OP66.02 |
| Salpingectomy with removal of a tubal pregnancy |  | OP66.62 |
| Removal of an ectopic pregnancy |  | OP74.3 |
| Post-ectopic depression |  | 296.2, 296.3, 296.5, 300.4, 309.0, 309.1, 309.28; Within 4 weeks after ectopic pregnancy |
| Antidepressants |  | Agomelatine, TCA. SSRI, RIMA, NDRI, SNRI |
| Agomelatine |  | N06AX22 |
| Tricyclic and tetracyclic antidepressants | TCA | Amitriptyline, Clomipramine, Doxepin, Imipramine, Maprotiline |
| Amitriptyline |  | N06AA09 |
| Clomipramine |  | N06AA04 |
| Doxepin |  | N06AA12 |
| Imipramine |  | N06AA02 |
| Maprotiline |  | N06AA21 |
| Selective serotonin reuptake inhibitors | SSRI | Citalopram, Escitalopram, Fluoxetine, Fluvoxamine, Paroxetine, Sertraline |
| Citalopram |  | N06AB04 |
| Escitalopram |  | N06AB10 |
| Fluoxetine |  | N06AB03 |
| Fluvoxamine |  | N06AB08 |
| Paroxetine |  | N06AB05 |
| Sertraline |  | N06AB06 |
| Reversible Monoamine oxidase inhibitors | RIMA | Moclobemide |
| Moclobemide |  | N06AG02 |
| Norepinephrine-Dopamine Reuptake inhibitor | NDRI | Bupropion |
| Bupropion |  | N06AX12 |
| Serotonin-Norepinephrine Reuptake inhibitor | SNRI | Duloxetine, Venlafaxine, Milnacipran |
| Duloxetine |  | N06AX21 |
| Venlafaxine |  | N06AX16 |
| Milnacipran |  | N06AX17 |
| Stroke |  | 430 - 438 |
| **Comorbidities:** Charlson comorbidity index revised | CCI_R | CCI removed dementia |

**ICD-9-CM: International Classification of Diseases, 9th Revision, Clinical Modification; ATC-Code: Anatomical Therapeutic Chemical Codes; TCA: Tricyclic Antidepressants; SSR: Selective Serotonin Reuptake Inhibitors; RIMA: Reversible Monoamine Oxidase Inhibitors; NDRI: Norepinephrine-dopamine reuptake inhibitors; SNRI: Serotonin/Norepinephrine reuptake**

**inhibitor**

**Table S2 Age of dementia patients**

| **Ectopic pregnancy** | **Min** | **Median** | **Max** | **Mean ± SD** | ***P*** |
| --- | --- | --- | --- | --- | --- |
| With | 43.36 | 51.67 | 58.64 | 52.12 ± 8.94 |  |
| Without | 44.22 | 52.03 | 59.91 | 52.91 ± 9.07 |  |
| Total | 43.36 | 51.84 | 59.91 | 52.65 ± 9.03 | 0.045 |
| **SD: Standard Deviations*; P*: t-test** | | | | | |

Table S3 Factors of dementia subgroups by using Cox regression with / without Fine & Gray's competing risk model and Bonferroni correction for multiple comparisons

| **Ectopic pregnancy** | **With** | | | **Without** *(Reference)* | | | ***No competing risk*** | | | | ***Competing risk*** | | | |
| --- | --- | --- | --- | --- | --- | --- | --- | --- | --- | --- | --- | --- | --- | --- |
| **Dementia subgroups** | **Events** | **PYs** | **Rate** | **Events** | **PYs** | **Rate** | **aHR** | **95% CI** | **95% CI** | ***P*** | **asHR** | **95% CI** | **95% CI** | ***P*** |
| **Overall dementia** | 791 | 142,297.78 | 555.88 | 1,572 | 435,652.39 | 360.84 | 1.604 | 1.375 | 1.982 | < 0.001 | 1.644 | 1.394 | 2.053 | < 0.001 |
| AD | 42 | 142,297.78 | 29.52 | 134 | 435,652.39 | 30.76 | 1.184 | 1.016 | 1.468 | 0.042 | 1.219 | 1.035 | 1.522 | 0.033 |
| VaD | 568 | 142,297.78 | 399.16 | 891 | 435,652.39 | 204.52 | 1.803 | 1.545 | 2.229 | < 0.001 | 1.848 | 1.567 | 2.309 | < 0.001 |
| Others | 181 | 142,297.78 | 127.20 | 547 | 435,652.39 | 125.56 | 1.269 | 1.090 | 1.567 | 0.005 | 1.303 | 1.108 | 1.627 | < 0.001 |
| **PYs = Person-years, Rate: per 100,000 PYs, aHR = Adjusted Hazard ratio: Adjusted for the variables listed in Table 3, CI = confidence interval** | | | | | | | | | | | | | | |
| **asHR = Adjusted Subdistrubtion Hazard ratio: Adjusted for the variables listed in Table 3; Competing variable: all-cause mortality** | | | | | | | | | | | | | | |
